# Chemokine-Driven Migration of Pro-Inflammatory CD4^+^ T Cells in CNS Autoimmune Disease

**DOI:** 10.3389/fimmu.2022.817473

**Published:** 2022-02-16

**Authors:** Aaron H. S. Heng, Caleb W. Han, Caitlin Abbott, Shaun R. McColl, Iain Comerford

**Affiliations:** The Chemokine Biology Laboratory, Department of Molecular and Biomedical Science, School of Biological Sciences, Faculty of Science, The University of Adelaide, Adelaide, SA, Australia

**Keywords:** chemokine, migration, EAE (experimental autoimmune encephalomyelitis), multiple sclerosis, Th subsets

## Abstract

Pro-inflammatory CD4^+^ T helper (Th) cells drive the pathogenesis of many autoimmune conditions. Recent advances have modified views of the phenotype of pro-inflammatory Th cells in autoimmunity, extending the breadth of known Th cell subsets that operate as drivers of these responses. Heterogeneity and plasticity within Th1 and Th17 cells, and the discovery of subsets of Th cells dedicated to production of other pro-inflammatory cytokines such as GM-CSF have led to these advances. Here, we review recent progress in this area and focus specifically upon evidence for chemokine receptors that drive recruitment of these various pro-inflammatory Th cell subsets to sites of autoimmune inflammation in the CNS. We discuss expression of specific chemokine receptors by subsets of pro-inflammatory Th cells and highlight which receptors may be tractable targets of therapeutic interventions to limit pathogenic Th cell recruitment in autoimmunity.

## Introduction

Th cells play pivotal roles in tissue inflammation in a variety of human autoimmune conditions such as multiple sclerosis (MS), psoriasis and rheumatoid arthritis (RA). Various subsets of Th cells that produce distinct arrays of cytokines can either promote or inhibit autoimmune inflammation (1). Th subsets are well-recognised for their functional specialisation in polarisation of immune responses towards effector responses tailored for destruction/elimination of specific types of microbial assault. For example, intracellular microbes are combated by the actions of IFNγ-secreting Th1 cells; responses suited for expulsion of helminths and nematodes are triggered following activation of interleukin (IL)-4/5/13-secreting Th2 cells; and extracellular bacteria and fungus are controlled by phagocyte responses amplified by IL-17A/F-secreting Th17 cells. An underlying pathological driver of numerous chronic autoimmune diseases has been attributed to the actions of misdirected Th1 and/or Th17 responses. These subsets have been implicated to be drivers of tissue damage through their local production of pro-inflammatory cytokines in the local microenvironment that facilitates the persistent recruitment and activation of tissue-damaging myeloid cells, thus culminating in a chronic inflammatory response. When considering the pathogenesis of Th cell mediated tissue inflammation, an essential step is their recruitment from the peripheral circulation into sites of inflammation (2). Thus, it is of importance to elucidate and understand the molecular mechanisms pro-inflammatory Th cells use to traffic into tissues where they drive inflammatory disease. In doing so, molecular targets amenable to therapeutic intervention in autoimmunity may be realized.

### Pro-Inflammatory Th Cells

Prior to 2005, IFNγ-producing Th1 cells were considered the main CD4^+^ T cell subset responsible for driving autoimmune inflammation in the central nervous system (CNS). These cells were abundantly recruited to sites of autoimmune inflammation where they supported monocyte recruitment, macrophage activation and other cell-mediated effector responses. Th1 cells rely on IFNγ and IL-12 for their differentiation and are characterized by expression of the transcription factor T-Bet. Supporting their pro-inflammatory function in autoimmunity, studies using a mouse model of MS, experimental autoimmune encephalomyelitis (EAE), demonstrated that deletion of the p40 subunit of IL-12 or the IL-12Rβ1 chain rendered mice resistant to neuroinflammation with impaired Th1 responses (3). However, the discovery of the cytokine IL-23 and its receptor fundamentally altered the Th1-driven view of autoimmunity. IL-23 shares the p40 subunit of IL-12 and the IL-23 receptor also uses the IL-12Rβ1 chain (4, 5). Deletion of the IL-12p35 subunit or the IL-12Rβ2 chain, which are specific for IL-12, had no effect on EAE pathogenesis while loss of the IL-23p19 subunit or IL-23R chain resulted in resistance to EAE (3, 5, 6). Subsequent studies into the IL-23-dependent cells in EAE revealed a population of CD4^+^ T cells in the inflamed CNS that were predominantly IL-17A-producers (7). IL-23 signalling was also demonstrated to promote the emergence and expansion of IL-17A-secreting CD4^+^ T cells in both recently activated and memory CD4^+^ T cell compartments, and IL-23-driven MOG-reactive CD4^+^ T cells were shown to be sufficient in driving pathogenesis in adoptive transfer of EAE. Th17 cells quickly became established as a distinct lineage of Th cells (8, 9), with a wide variety of roles in adaptive immunity to extracellular microbes and strongly linked to pro-inflammatory responses in autoimmunity.

Th17 cells are characterised by secretion of IL-17A/F and the expression of the transcription factor retinoic acid receptor-related orphan receptor C (RORγt) (8, 10). These cells are primarily involved in the maintenance of mucosal barrier function and defence against extracellular pathogens (11, 12). During homeostasis, Th17 cells are present at mucosal surfaces to maintain tissue homeostasis and prevent microbial colonisation (13). Antigen-specific activation in the presence of IL-6 triggers differentiation of naïve T cells into Th17 cells *via* JAK1 and JAK2 signalling, which triggers activation of the STAT3 pathway (14, 15). Th17 cells produce their signature cytokines IL-17A, IL-17F, IL-21 and IL-22, which are involved in host mucosal defence against extracellular pathogens by inducing chemokine production for increased neutrophil recruitment and enhancing expression of antimicrobial peptides. IL-17A has been implicated in an array of autoimmune diseases such as in MS, psoriasis, SLE, and RA. Engagement of IL-17A with the IL-17 receptor activates the NFκB pathway, which stimulates production of proinflammatory cytokines including IL-1β, GM-CSF and chemokines such as CCL2 (16), CXCL1 (17) and CCL20 (18). These cytokines and chemokines act in concert to accelerate the inflammatory response and to direct neutrophils and monocytes to the site of inflammation. An interesting characteristic of Th17 cells is their context-dependant plasticity, allowing Th17 cells to transdifferentiate and reprogram for production of a different array of cytokines in response to signals from antigen-presenting cells (APCs) (19–25). For example, studies have shown that Th17 cells transdifferentiate into IFNγ-producing Th1-like cells, IL-4-producing Th2-like cells, type 1 regulatory cell (Tr1)-like cells and IgA -promoting T follicular helper-like cells (26–28). The reverse has also been shown to be possible to a certain extent. Studies have shown that Th1-like cells that emerge from Th17 cells are able to transdifferentiate back into Th17 cells, and that FOXP3^+^ T cells are able to transdifferentiate into Th17 cells in a model of RA (29, 30). Th17 cells can also turn off production of IL-17A, transdifferentiating into so called ‘exTh17’ cells (31). This plasticity appears to give Th17 cells a degree of flexibility that allows them to appropriately respond to an evolving antigenic threat by either amplifying, attenuating, or modulating the inflammatory response.

In EAE, neutralisation of IL-17A was reported to delay the onset of clinical disease (32), however multiple studies have also concluded that IL-17A is not essential for EAE pathogenesis (33–35). This has prompted further study of the Th17-derived cytokines that drive EAE. These studies have identified GM-CSF as being a critical T cell-derived cytokine for the pathogenesis of classical EAE (35–37). GM-CSF-deficient mice are highly resistant to EAE induction (38), and GM-CSF-deficient myelin-specific T cells are unable to transfer disease (35). Furthermore, dysregulated overexpression of GM-CSF in polyclonal Th cells was sufficient to induce neuroinflammation by promoting an influx of phagocytes into the CNS (39). T cell-derived GM-CSF was more specifically shown to act on a variety of myeloid subsets including inflammatory CCR2^+^Ly6C^hi^ monocytes, monocyte-derived dendritic cells (moDCs) and microglia, all of which have been previously ascribed pathogenic roles in EAE *via* GM-CSF-dependent signalling (40). Moreover, loss of the GM-CSF receptor on myeloid cells rendered mice resistant to EAE induction (40, 41). While most studies indicate that GM-CSF is essential for inducing EAE pathogenesis, two studies have disputed this claim by showing that EAE can be induced *via* both direct and indirect immunisation of GM-CSF-deficient mice (42, 43). Despite these findings, both studies showed that GM-CSF is a potent driver of inflammation during EAE.

Mechanisms driving the conversion of IL-17-secreting Th17 cells to proinflammatory GM-CSF-secreting Th17 or exTh17 cells *in vivo* have been elucidated to some extent. IL-23 and IL-1β have been demonstrated to induce a more inflammatory GM-CSF/IFNγ cytokine production profile in Th17 cells *in vitro* and have also been shown to have indispensable roles in the pathogenesis of EAE in knock-out studies. Studies have also shown that Th17 cells generated in the presence of TGFβ3 acquired inflammatory capacity after prolonged exposure to IL-23, upregulating IFNγ and adopting a Th1-like phenotype (44, 45). TGFβ3 restrains IL-10 production and supports the production of proinflammatory cytokines by Th17 cells through induction of Smad1 and Smad5 (46). Additionally, IL-7, which is expressed highly in the CNS of mice with EAE, has also been implicated in the induction of GM-CSF production and acquisition of a more Th1-like phenotype by Th17 cells (47, 48). Although it has been shown that IL-7Ra expression is induced by IL-23 (49), IL-7R signalling requires IL-23 responsiveness in Th17 cells to promote their pathogenicity in EAE (47). However, recent studies suggests that IL-7 may be involved in the generation or maintenance of memory Th17 cells at the site of inflammation (50). Interestingly, a recent study using a reporter and fate mapper of GM-CSF showed that Th cells that had produced GM-CSF have a distinct epigenetic profile indicative of long-term stability (51). It is possible that IL-7 contributes to stability of GM-CSF-secreting cells, but this remains to be tested. Furthermore, Th17 cells in the CNS have the highest expression of IL-7R followed by Th1-like Th17 cells and then Th1 cells, which express the lowest levels of this receptor (47). This further implicates a role for IL-7 signalling in the expansion or maintenance of exTh17 cells in the CNS.

Recent studies have identified another subset of Th cells that abundantly produces GM-CSF and low amounts of cytokines associated with other Th subsets (52). These cells, dubbed ThGM cells, are believed to be a distinct Th subset from Th1 or Th17 cells as they do not require the expression of RORγt or T-Bet to support high levels of GM-CSF production, and unlike Th1 and Th17 cells, which develop *via* a STAT4 and STAT3 pathway respectively, they have been shown to derive from naïve CD4^+^ precursors that activate the STAT5 pathway following IL-7 and/or IL-2 stimulation (48). In support, evidence implicates STAT5 as being an important factor in EAE. Mice harbouring STAT5-deficient CD4^+^ T cells are less susceptible to EAE compared to WT, and this was associated with reduced GM-CSF production without impacting IFNγ or IL-17. This is further supported by studies that demonstrated that GM-CSF-producing CD4^+^ T cells generated through an IL-7/STAT5 pathway were more encephalitogenic in an adoptive EAE model compared to Th17 or Th1 cells (48).

## Migratory Mechanisms of Autoreactive Th Cells in EAE/MS

**Figure 1:**
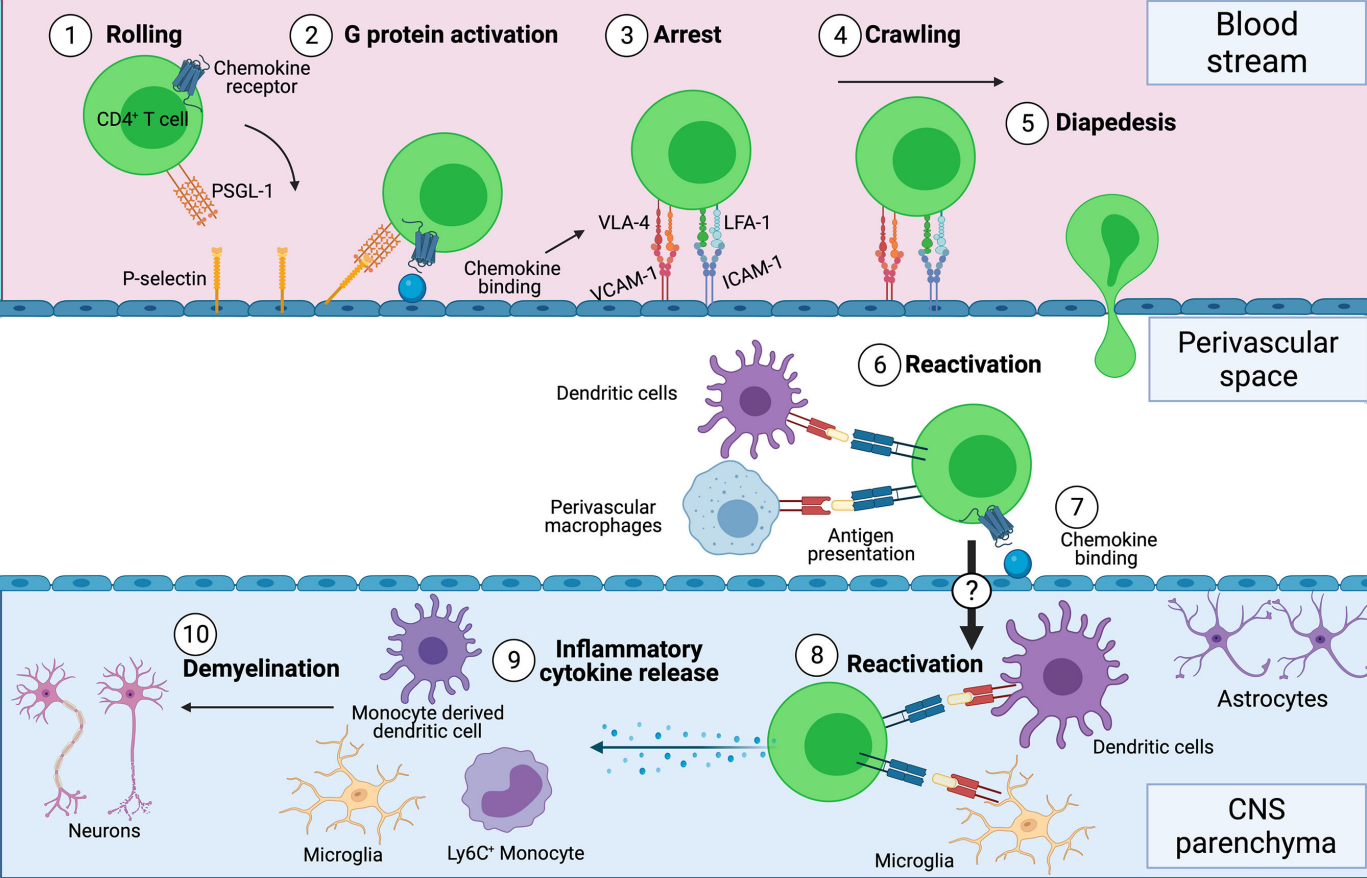
T cell recruitment into the CNS during neuroinflammation. (1) After activation in the periphery, CD4^+^ T cells roll across the surface of the blood vessel in a process mediated by P-selectin and PSGL-1 interactions. (2) Binding of chemokine receptors to chemokines expressed on the surface of the endothelial cells activate G-proteins which trigger conformational changes of the integrins VLA-4 and LFA-1, allowing for arrest (3) of the T cells to VCAM-1 and ICAM-1 respectively. (4) T cells crawl across the endothelial cells, (5) undergo diapedesis and eventually enter the perivascular space. (6) Perivascular macrophages and dendritic cells reactivate the T cells by presenting antigen, (7) which allows T cells to migrate into the CNS parenchyma. (8-10) Once in the parenchyma, T cells can be reactivated by antigen presenting cells and release inflammatory cytokines such as GM-CSF which activates and recruits Ly6C^+^ monocytes and monocyte-derived dendritic cells that drive demyelination. Figure was created using Biorender.

The CNS has several unique barriers in place to maintain homeostasis and limit leukocyte infiltration. To initiate CNS autoimmunity, autoantigen-specific T cells are activated in the periphery and travel to the CNS, where they are reactivated by APCs presenting the autoantigen. This triggers the release of cytokines to activate and recruit other inflammatory cells to cause inflammation. However, the CNS employs various obstacles such as the blood brain barrier (BBB) and the blood cerebral spinal fluid (CSF) barrier (BCSFB) to limit access of T cells [reviewed in (53)]. In order to breech these barriers, the coordinated use of selectins, integrins and chemokines are required for efficient T cell migration into the CNS (depicted in **Figure 1**).

### Selectins

Selectins are a family of transmembrane C-type lectins expressed by bone marrow-derived cells and endothelial cells (54). The selectin family consists of three members: L-selectin, P-selectin, and E-selectin. While selectins and their ligands are clearly involved in T cell rolling on cerebrovascular endothelial cells, a clear picture of the essential selectin-mediated signals required for T cell entry to the CNS has yet to emerge and differences in selectin usage by distinct subsets of Th cells remains to be determined. L-selectin is expressed on many subsets of T cells, including naïve and some memory T cells where it is required for homing to lymph nodes. While studies have also implicated L-selectin in the pathogenesis of EAE/MS (55, 56), this appears to be mainly due to the role of this adhesion molecule in T cell priming rather than their effector functions (57). PSGL-1 and its ligand P-Selectin have clearly been shown to mediate T cell rolling on BBB microvasculature (58, 59), however this interaction between P-selectin and PSGL-1 is evidently dispensable to T cell recruitment to the CNS and for EAE pathogenesis (60). More recently the P-selectin ligand TIM-1 was implicated as the key adhesive signal that mediated Th1 and Th17 rolling on brain microvasculature (61). Another recent study has implicated the E-selectin ligand CD43 on T cells as a key mediator of Th17 adhesion to the brain endothelial cells, but this appears to be independent of interactions with E-selectin (62). Taken together, there is still much to learn about the essential early steps in pathogenic T cell rolling on brain vasculature and which selectin molecules, or other interactions, mediate this process in distinct subsets of these cells.

### Integrins

Integrins have been long implicated in recruitment of CD4^+^ T cells to the CNS during autoimmunity. For example, the interaction between the α4β1 integrin (CD29/CD49d, VLA-4) on effector T cells and its ligand VCAM-1 on BBB endothelial cells has been implicated in early entry of T cells into the CNS. Neutralisation of the α4 integrin inhibits both EAE pathogenesis and prevents encephalitogenic T cell recruitment into the CNS parenchyma (63–65). These discoveries led to the development of Natalizumab, an anti-α4 integrin blocking monoclonal antibody, now used for the treatment of MS. Natalizumab displays significant clinical efficacy in reducing relapses and disease progression in relapsing-remitting MS patients (66–69). While blocking of α4 integrin clearly inhibits EAE and MS pathogenesis, the specific role of this integrin in the recruitment of pro-inflammatory T cell subsets to the CNS is more complex. VLA-4 is strongly implicated in monocyte and Th1 cell adhesion to the inflamed BBB, but Th17 recruitment to the brain during EAE appears to be entirely independent of this integrin (70, 71). Instead, Th17 cells appear to be more reliant on the αLβ2 integrin (LFA-1) for entry into the CNS (71), a characteristic they also appear to share with Tregs (72). In addition to mediating recruitment to the inflamed CNS, recent 2-photon imaging studies have also implicated the αLβ2 integrin in regulating both Th1 and Th17 motility within the CNS, and blocking this integrin using intrathecal anti-LFA-1 antibodies substantially attenuated disease pathogenesis in EAE mice (73).

Other T cell expressed integrins have also been implicated in recruitment to the inflamed CNS. The integrin ανβ3 was found to be expressed specifically by Th17 cells, and was upregulated through IL-23 signalling (74). This identified that ανβ3-expressing Th17/exTh17 cells increased in number during EAE, and deficiency of this integrin using KO mice or using an inhibitor severely dampened Th17-mediated EAE, while not affecting Th1-mediated EAE. Transfer of ανβ3-deficient Th17 cells also resulted in reduced severity of Th17-mediated EAE. Interestingly, this study suggested ανβ3-deficient Th17 cells were unable to infiltrate the CNS, pointing to a role of ανβ3 in allowing for pathogenic Th17 cell migration to the CNS during EAE (74). In support of this finding, another study using an ανβ3 binding peptide also showed efficacy in reducing clinical signs of EAE in rats (75).

Together, while α4 integrins have been shown to be involved in T cell recruitment to the CNS EAE, other integrins such as ανβ3 and αLβ2 likely also contribute to the migration of pathogenic Th cells into the CNS. It is probable that these integrins play a coordinated role in influencing the trafficking of Th cells and is therefore likely to be able to compensate for the loss of either integrin. Therefore, future research into elucidating the exact roles these integrins play during EAE, as well as the location these integrins can allow Th cells to enter the CNS should be investigated. Doing so will undoubtably uncover new insights into the properties and process of pathogenic T cell migration during CNS autoimmunity. The remainder of this review will now focus on the chemokine receptors that control the mobility of autoinflammatory Th cells, as well as their impact in the pathogenesis of EAE.

### Chemokine Receptor-Driven Migration of Th Cells to the CNS in EAE/MS

Chemokines and their associated receptors are key modulators of T cell migration. Chemokines are a large group of small structurally related cytokines that function as chemoattractants and play a fundamental role in T cell biology by directing cell migration through interactions with chemokine receptors [reviewed in (76, 77)]. Chemokine receptors are G-protein coupled receptors that upon ligation trigger intracellular signalling cascades resulting in leading edge polarisation, integrin activation and ultimately directed cell migration towards extracellular sources of chemokines (78). This allows for T cell traffic to specific microanatomical sites and/or areas of inflammation. Differential chemokine receptor expression profiles of Th subsets functionally distinguish them according to their differing circulation patterns and migratory potential, and chemokine receptor profiling has been used to characterise and identify specific populations of Th cells (79, 80). For example, the expression of the chemokine receptor CCR7 is typically present on naïve CD4 T cells and some memory Th cells to allow them to home towards the chemokines CCL19 and CCL21 within T cell areas of the secondary lymphoid organs, where they encounter mature APCs (81). In contrast, activated Th cells downregulate CCR7 and express an array of inflammatory chemokine receptors (such as CXCR3, CXCR6, CCR2, CCR4, CCR5, CCR6) that act in concert to drive recruitment to inflamed tissues where ligands for these receptors are produced in response to tissue damage or infection (80, 82, 83). Th1 cells are well known to express CXCR3 that directs these cells to sites of production of CXCL9/10/11, which are induced by type-I interferon signalling (84, 85). Th17 cells are known to express CCR6 (21, 86, 87), which along with its sole cognate chemokine ligand CCL20, plays a role in both homeostasis and inflammation (88). Under resting conditions, constitutive CCL20 expression in the intestinal mucosa attracts CCR6-expressing cells such as Th17 cells, B cells, dendritic cells (DC) and regulatory T cells (Treg), whereas under inflammatory conditions CCR6 recruits activated T cells, DCs and macrophages to areas of acute inflammation at epithelial sites (89, 90).

In EAE/MS, autoreactive T cells gain access to the CNS in spite of significant physical barriers in place to impede this process. The two main barriers that impede immune cell entry into the CNS are the BBB and BCSFB. Bypassing the BBB and BCSFB allows access to the perivascular space and the CSF subarachnoid space (SAS) respectively, whereby the cells can then access the CNS by bypassing the glia limitans from the perivascular space or through the CSF [reviewed in (91)]. The chemokines expressed in the CNS under non-inflammatory conditions to mediate initial recruitment of encephalitogenic Th cells in EAE are not yet fully understood, if indeed a chemokine-dependent signal is required. Candidate chemokines expressed in the non-inflamed CNS are CX3CL1, CXCL12, CCL20 and CCL21 (92–98). While the CX3CL1 receptor CX3CR1 clearly impacts on CNS autoimmunity through effects on myeloid cells, its role in T cell recruitment to the brain during neuroinflammation is controversial with one study indicating no T cell intrinsic role for CX3CR1 in EAE and another study showing CX3CR1-dependent recruitment of T cells to the brain during neuroinflammation (99, 100). CXCL12 is expressed along the basolateral surfaces of CNS endothelial cells (101). Here, it appears retain T cells, which almost ubiquitously express CXCR4, that have crossed the BBB to the perivascular space and CXCR4 limits their infiltration of the CNS parenchyma. The role of CCL21/CCR7 in T cell entry to the uninflamed CNS has not been fully explored but clinical EAE does not appear to be affected by whole animal deletion of CCR7 (102); and a study where CCR7-deficiency was limited to T cells imply that this receptor is involved in T cell priming during EAE but not entry to the CNS (103). However, a recent study administration of a dietary flavonoid downregulated CCR7 on CD4^+^ T cells in the CNS, thus reducing infiltration of pathogenic T cells into the CNS and thus reducing EAE severity (104). CCL20 is constitutively expressed on the epithelial cells of the choroid plexus, and may be the chemokine signal that allows CCR6^+^ T cells to bypass the BCSFB (95). Another study by Murakami and colleagues indicated that CCL20 was expressed by dorsal blood vessels in the 5^th^ lumbar spinal cord where it is induced by IL-6 and that this provides a site of pathogenic T cell recruitment into the CNS in EAE (105).

The molecular mechanisms governing how GM-CSF-secreting exTh17 cells or ThGM cells infiltrate the CNS have yet to be revealed. While it has been shown that CCR6^+^ Th17 cells infiltrate the choroid plexus early during the initial stages of EAE, where they are reactivated by local APCs (95, 106), not much is known about the mechanisms used by pathogenic Th subsets to enter the CNS during the later phases of EAE. It is possible that cytokines released by early infiltrating Th17 cells (such as TNFα or IL-17A) disrupt BBB integrity and then allow cells expressing chemokine receptors such as CCR2 or CXCR6 to enter the CNS directly from brain capillaries (107, 108). Alternatively, pathogenic T cells could manoeuvre into the CNS *via* expression of other chemokine receptors that may allow entry through the BBB or BCSFB. Expression of chemokines such as CXCL16 and CXCL12 are upregulated in the inflamed CNS (109, 110), and whether these chemokines are involved in recruiting T cells such as ThGM cells into the CNS remains an interesting area for future research.

**Figure 2:**
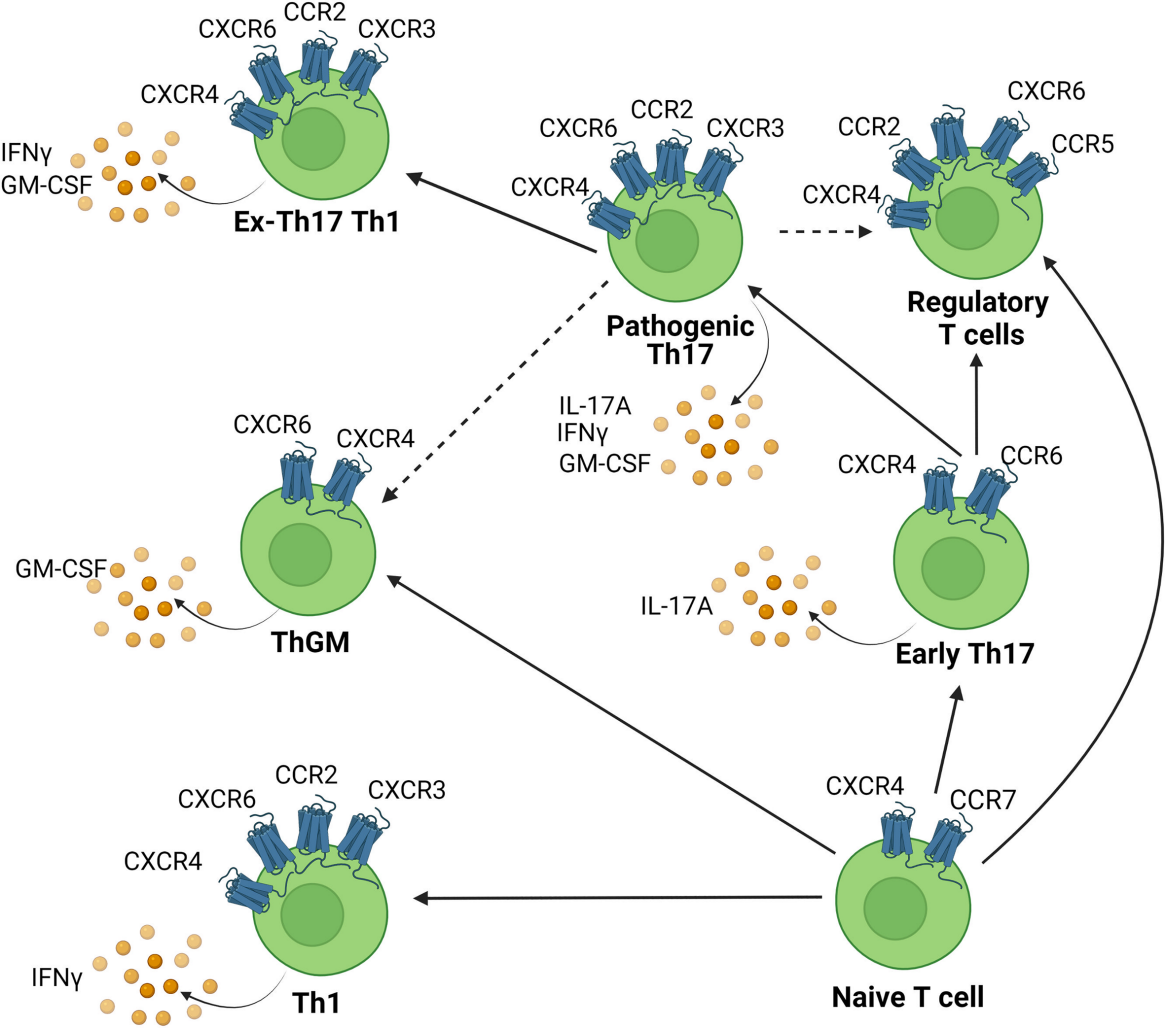
Chemokine receptor expression on CNS infiltrating Th cell subsets. Chemokines attract chemokine receptor expressing Th cells to infiltrate the CNS. Various Th subsets are capable of migration into the CNS to cause inflammation. This includes Th1, Th17, ThGM cells and each are associated with distinct chemokine receptor signatures. Solid lines indicate pathways of cellular transdifferentiation. Dotted lines indicate potential pathways that remain to be elucidated. Figure was created using Biorender.

It has been shown that various chemokine receptors are involved during the pathogenesis of EAE/MS. These receptors are expressed on various T cell subsets involved in EAE such as Th17 cells and ThGM cells and their expression is regulated temporally during EAE. Th17 cells initially express CCR6 but can switch chemokine receptor expression and upregulate other receptors such as CCR2, which reflects an increase in their pathogenic signature (111). GM-CSF-producing cells, including Th17, exTh17 cells, and ThGM cells also potentially utilise these receptors to infiltrate the CNS. The chemokine receptor expression profile of the various subsets of Th cells implicated in EAE and the relationship between these cell types is depicted in **Figure 2**. We will now discuss each of the main candidate chemokine receptors that appear likely to contribute to pathogenic T cell recruitment to the CNS in detail.

### CCR6

Th17 cells have been extensively shown to express CCR6 (21, 86, 87), the sole receptor for the chemokine CCL20 (88, 112). Studies have also reported increased expression of CCL20 in the draining lymph nodes (dLN) and spinal cord of mice following immunisation for EAE (113, 114). CCL20 is also expressed in the CNS by astrocytes (115), and decreased production by astrocytes is correlated with a reduced clinical disease in EAE (116). Furthermore, CCL20 also appears to be involved in Th17 cell egress from the LN, prior to migration into the CNS (114). Neutralizing either CCL20 or CCR6 in EAE mice decreased disease severity in various studies, highlighting the role of the CCL20/CCR6 axis in EAE (113, 114, 117). A seminal study by Reboldi et al., demonstrated that Th17 cells migrate through the blood-CSF barrier into the uninflamed brain through the interaction of CCR6 with CCL20 expressed on the epithelial cells of the choroid plexus (95). This study also showed that *Ccr6* ^-/-^ mice were resistant to the induction of EAE and that transfer of CCR6-sufficient T cells restored susceptibility of these mice to EAE. It was noted that the subsequent recruitment of Th17 cells into the CNS was largely CCR6-independent, which established a model that CCR6 coordinates the first wave of Th17 cells required to initiate inflammation in EAE but is dispensable for the subsequent infiltration of autoreactive cells into the inflamed CNS.

Since then, various studies have shown that CCR6 signalling does not inherently drive pathogenic T cell recruitment in EAE, as *Ccr6^-/-^* mice showed delayed disease onset but eventually develop disease with increased severity compared to WT controls (118, 119). This highlights the essential role of other chemokine receptors or migratory factors that can compensate for the loss of CCR6 for recruitment of inflammatory T cells to the CNS. Furthermore, Treg cells also express CCR6, and have been shown to migrate to the CNS *via* CCR6 to control inflammation during EAE (117). In addition, exTh17 cells that have transitioned to a Th1-like phenotype were found to downregulate CCR6, suggesting that pathogenic cells associated with driving EAE utilise a different mode of migration (31). In support, a study from our laboratory (111) found that Th17 cells undergo an IL-23-dependent switch of chemokine receptor expression during EAE, which involves downregulation of CCR6 as they increase pro-inflammatory GM-CSF and IFNγ production. In humans, CCR6 is commonly used in combination with CXCR3 to identify Th17 cells (120, 121). While a CCR6^+^CXCR3^-^ profile enriches for Th17 cells, Th17 cells that co-express IFNγ have been found to also express CXCR3 (122). These CCR6/CXCR3 double expressing Th17 cells (dubbed Th17.1 or non-classic Th1) have also been associated with MS disease severity (123, 124).

From the discussion above, while CCR6/CCL20 clearly play a role in EAE/MS, there are other factors that must be considered with regards to their potential as therapeutic targets to treat MS. These includes the likely effectiveness of disrupting CCR6 activity, which could affect the trafficking of Tregs or other regulatory leukocyte populations into the CNS, as well as having no effect on inflammatory T cell subsets that have downregulated CCR6 or do not express this receptor.

### CCR2

CCR2 is involved largely in monocyte and macrophage mobilization (125–127) but is also expressed by activated T cells, astrocytes, microglia cells, and has been shown to be expressed by infiltrating autoinflammatory Th cells in the CNS during MS/EAE (111, 128–130). CCR2 is potently activated by the chemokine CCL2, which has been shown to be expressed by astroglial cells in EAE (131, 132). Multiple studies have demonstrated significant protective effects of CCR2-deficiency in EAE, as CCR2-deficient mice are resistant to EAE induction, and neutralising antibodies against CCL2 reduced EAE severity (133–135). This has been proposed to be due to the effects on CCR2^+^ Ly6C^hi^ monocytes, which have been shown to be important in mediating tissue inflammation during neuronal autoimmunity through a GM-CSF-dependent pathway (40, 127). A study which transferred primed MOG-specific T cells from WT or CCR2-deficient mice into WT or CCR2-deficient hosts showed that while *Ccr2^-/-^* T cells were able to transfer disease to a WT mice, primed T cells from WT mice were unable to do so when transferred into *Ccr2^-/-^* mice (134). This suggest that the major role for CCR2 in EAE was extrinsic to antigen-specific T cells. Similar results were found in *Ccl2^-/-^* mice, which had delayed onset of EAE, but were able to generate Th cells that could transfer disease into WT mice (136). Another study looking at depletion of CCL2 from astrocytes in EAE mice reported delayed onset and severity of disease, but also discovered an increase in Th17 cell infiltration in the CNS along with increased levels of *Il6* mRNA (132). This suggests that in the absence of astroglial CCL2, either an increase in other chemotactic factors allows for an increase in Th17 cells migrating in, or that astrocytes increased production of IL-6 which resulted in an increased amount of Th17 differentiation in the CNS. A recent study that silenced activity of excitatory neurons in EAE resulted in decreased expression of *Ccl2* and *Ccr2* mRNA in EAE lesions, suppressing migration of Th cells and alleviating motor deficits of EAE (137). Interestingly, a study has shown that blocking CCR2 can lead to an increase in severity of experimental arthritis, which is in contrast to the proinflammatory nature of CCR2 (138). This has been explained to be due to the inability of CCR2-expressing Tregs to migrate to the affected areas to regulate the inflammation.

CCR2 and CCL2 have also been implicated with MS disease severity. CCR2 has been found to be highly expressed by infiltrating T cells within and around active MS lesions, and CCL2 is expressed by astrocytes within lesions (115, 129, 139, 140). As such, CCR2/CCL2 targeting drugs have been explored as treatments for MS and entered as candidates in clinical trials. While early trials reported a good safety profile of the drug candidates, they failed to induce significant therapeutic benefits and the trials were abandoned (141, 142). The lack of efficacy of CCR2 antagonists in MS patients may stem from complex compensatory mechanisms T cells have to traffic into the CNS during MS. Thus, inhibition of CCR2 function alone would thus be unable to deter T cell infiltration. In support of this, a recent study demonstrated that Clozapine, a commonly used drug in MS patient treatment, reduced disease onset and severity in EAE by attenuating migration of immune cells including T cells by specifically reducing both CCL2 and CCL5 levels (143). Our chemokine receptor expression profiling of Th17 cell indicated that Th17 cells express both CCR6 and CCR2 in EAE mice. Over the course of the model, Th17 cells exhibited a temporal transition from CCR6 to CCR2 expression, which was associated with a more inflammatory IFNγ/GM-CSF-producing state (111). Moreover, mice lacking CCR2 specifically in the T cell compartment and transfer of *Ccr2*-deficient T cells showed reduced EAE and fewer GM-CSF-secreting Th17 cells in the CNS compared to controls. However, mice with a T cell compartment deficient in CCR2 were still able to induce EAE after a delay, suggesting that inhibiting CCR2 is not essential for all autoreactive T cell entry into the CNS. Therefore, other migratory factors aside from CCR2 on T cells should be explored as potential targets.

### CXCR3

CXCR3 has been extensively studied with regards to its role in inflammatory T cell recruitment during neuroinflammation. This receptor is abundantly expressed on CNS-infiltrating T lymphocytes from MS patients (144) and co-ordinates migration in response to its three ligands, CXCL9, CXCL10 and CXCL11 (145–147). Commonly associated with IFNγ-producing Th1 cells and CTLs, CXCR3 is expressed on activated and memory T cells and is induced after exposure to IFNγ (148, 149). Some Th17 cells also express CXCR3, and it has been shown to mediate migration of these cells in models of autoimmunity (150–152). Studies of the role of CXCR3 in EAE studies have yielded divergent results. Contrary to the expectation that CXCR3 would play a role in mediating migration of pathogenic Th subsets into the CNS, disrupting CXCR3 using antibodies or using *Cxcr3^-/-^* mice revealed that lack of CXCR3 signalling renders mice more susceptible to EAE (153–156). A study using anti-CXCR3 neutralising antibodies in rats showed that while there are high levels of CXCR3^+^ infiltrating T cells in the CNS of EAE rats, CXCR3 was redundant for T cell infiltration of the CNS (153). This study also showed that while anti-CXCR3 treatment was strongly effective against adoptive transfer of MBP-specific T cells, it did not affect active immunisation. This could be explained by active immunisation eliciting multiple arms of the immune response, which triggers activation of APCs leading to production of additional CXCR3-independent migratory factors in the CNS, thus allowing T cells recruitment to the CNS without CXCR3 function in this setting. Studies using *Cxcr3^-/-^* mice also showed that these mice did not differ from WT in terms of the amount or quality of infiltrating leukocytes during peak EAE. Instead, *Cxcr3^-/-^* mice exhibited severe BBB disruption, decreased levels of IFNγ, increased demyelination and axonal damage (155, 156). While the number of CD4^+^ T cells infiltrating the CNS was similar between *Cxcr3^-/-^* and WT mice, FOXP3^+^ regulatory T cells were significantly reduced in *Cxcr3^-/-^* mice, which may contribute to exacerbated disease. To add to the complexity regarding the role of CXCR3 in EAE, a study by Chung et al., showed that *Cxcr3^-/-^* mice were more susceptible to both actively and passively induced EAE compared to WT mice (154). This study highlighted that activated glial cell numbers are increased in *Cxcr3^-/-^* mice, and these *Cxcr3^-/-^* glial cells produce cytokines that promote Th17 cells, as mRNA levels of IL-6, IL-23p19 and IL-17A were all increased in glial cells in the absence of CXCR3. These findings correlated with an increased number and proliferative capabilities of Th17 cells in the CNS. This points to an indirect role of glial cell-derived CXCR3 in controlling Th17 levels in the CNS and hence restraining EAE pathogenesis in CXCR3-sufficient mice.

Contrary to studies that show that CXCR3 inhibits EAE pathogenesis, our previous studies have shown that administration of a truncated CXCL11 antagonist of CXCR3 prevented infiltration of CD4^+^ T cells into the CNS of mice with relapsing-remitting EAE and resulted in reduced disease severity (157). We found that CXCR3 antagonism blocked adoptive transfer of EAE but did not impact on T cell priming. These findings are consistent with the findings from Sporici and Issekutz in rat models of EAE where CXCR3 antibody blockade affects passively transferred EAE (153). In further support of this, Fife et al. reported that CXCL10 administration induced infiltration of CXCR3^+^ T cells into the CNS (158). A study by Schmitz et al. also reported a reduction in EAE clinical scores on *Cxcr3^-/-^* mice, which was associated with a reduction in IL-17A levels as compared to WT mice (159). Altogether, these studies point towards a complex role for CXCR3 in EAE. While CXCR3 signalling clearly can have suppressive effects on EAE pathogenesis, there is strong evidence that it is indeed used by subsets of CD4^+^ T cells to invade the CNS and this includes in some settings inflammatory CD4^+^ T cells that drive the disease. This requires future investigation with more targeted analysis of the known inflammatory subsets of T cells that have been identified in recent years. Of note, adoptive transfer of WT and *Cxcr3^-/-^* highly polarised encephalitogenic Th1 cells has clearly shown that recruitment of these cells to the CNS is independent of CXCR3 (160), and this also seems to be the case with adoptively transferred polarised Th17 cells (154).

In humans, CXCR3 levels are increased in the CSF and brain lesions of MS patients (161, 162). The levels of CXCL10 was also found to increase during relapses, and is expressed by macrophages and astrocytes in demyelinating lesions (162, 163). Moreover, treatment with IFNβ, which is an approved therapy for treatment of MS (164), specifically decreased CXCR3 expression levels on CD4^+^ T cells (165). As such, there seems to be compelling evidence that CXCR3 is involved in MS development and hence therapeutics targeting it may be beneficial for managing MS. However, there has only been one CXCR3 antagonist that has entered clinical trial for the treatment of psoriasis, and has been withdawn due to a lack of efficacy (166). However, as discussed below, there may still be utility in targeting CXCR3, particularly if this is in combination with other key migratory receptors and in contexts where this system is known to be driving pathogenesis.

### CCR5

Another chemokine receptor associated with T cell recruitment and MS/EAE pathogenesis is CCR5. Widely known for its role as an HIV-1 co-receptor, CCR5 has been implicated in T cell trafficking in various diseases including MS (167, 168). The ligands for CCR5, (in humans CCL3, CCL3L1, CCL4, CCL4L1, CCL5, CCL8 and CCL16) have been shown to be produced by cells in the CNS including microglia and astrocytes and may possibly play a role in attracting T cells into the CNS (169–171).

CCR5-expressing T cells were increased in the peripheral blood and demyelinating lesions of MS patients as compared to healthy patients and were also found in the brains of MS patient cadavers (168). CCR5^+^ T cells were also reported to produce more IFNγ than other T cells, consistent with a Th1-like phenotype. A study by Sato and colleagues reported that a subset of CCR2^+^CCR5^+^CCR6^-^ CD4^+^ T cells were enriched in the CSF of patients with severe MS compared to patients with other neurologic diseases (129). Furthermore, these cells produced various molecules that influence neuroinflammation, such as IFNγ and IL-17A, matrix metalloproteinase-9 (MMP9), and osteopontin. MMP9 has been shown to play a role in BBB disruption during EAE/MS (172, 173), while osteopontin is a phosphoprotein which has been associated with MS/EAE severity (174–176). This study also showed an increased potential of CCR2^+^CCR5^+^CCR6^-^ CD4^+^ T cells to traffic into the brain parenchyma, possibly due to their increased production of MMP9 and osteopontin (129).

In EAE, a clear picture of the role of CCR5 has yet to emerge. A study using mice deficient in either CCR5 or CCL3 reported that these mice were susceptible to EAE induction and were very similar to WT mice (177). However, another study recently reported that *Ccr5^-/-^* mice were resistant to EAE induction, displaying lowered disease severity than WT mice (178). Lower clinical disease was linked to several factors in that study, including impaired infiltration of leukocytes into the CNS, attenuated activation of astrocytes and microglial cells, and decreased levels of cytokines such as IL-1β in the spinal cord of *Ccr5^-/-^* mice. In support of a role of CCR5 in driving EAE, several studies have shown that CCR5 antagonists reduce migration of inflammatory cells into the CNS, resulting in reduced disease incidence, delayed onset, and severity in EAE mice (179–182). Furthermore, a study that neutralised the ligands of CCR5 ameliorated ongoing EAE (183). However, it should be noted that CCL3 and CCL5 are also ligands for CCR1 and CCR3, and hence the effect shown by this study was not necessarily a result of impairment of CCR5 activity. A recent study determining the effect of the Let-7 microRNAs determined that expression of Let-7 microRNA inhibited expression of CCR5 and CCR2 in pathogenic Th17 cells, leading to fewer numbers of infiltrating CD4 T cells in the CNS and reduced EAE pathogenesis (184). Intriguingly, CCR5, together with CXCR3, appear to contribute to adhesion to leptomeningeal structures and thus influence migration of T cells from the CSF into the CNS (185).Together, the evidence strongly suggests that CCR5 has a role in mediating the migration of autoinflammatory cells into the CNS during EAE, but whether CCR5 plays a prominent role in migration of recently identified inflammatory GM-CSF-secreting Th subsets will require further studies and use of experimental systems that can reveal the cell-intrinsic role of this receptor in pathogenic Th17 cells.

### CXCR6

Initially classified as a co-receptor for allowing the viruses SIV and HIV to enter cells (186), CXCR6 has been shown to be expressed on CD4^+^ T cells and is involved in the homing of CD4^+^ T cells to tissue sites of inflammation such as joints of RA patients (187, 188). Interestingly, CXCR6 has been reported to be highly expressed on IFNγ-producing and GM-CSF-producing CD4^+^ T cells (51, 189). The ligand for CXCR6, CXCL16, is a unique chemokine that acts both as an adhesion molecule to bind CXCR6-expressing cells and is cleaved as a soluble protein that mediates chemotaxis (190, 191). Membrane-tethered CXCL16 is also a scavenger receptor for oxidized low-density lipoprotein (192). CXCR6/CXCL16 have been implicated in various diseases, such as cancer, liver diseases and atherosclerosis (188, 193, 194). Expression of CXCL16, while low in the uninflamed brain, is drastically increased during inflammation as astrocytes and glial cells upregulate CXCL16 during neuroinflammation, and mRNA levels of *Cxcl16* have been shown to greatly increase in the brain and spinal cord during EAE (195, 196). Being selectively expressed on APCs such as DCs, CXCL16 has been shown to play a role in mediating the interactions between T cells and APCs in the splenic red pulp, allowing for the recruitment and activation of T cells and NKT cells (189, 197, 198). CXCR6 is used as a marker of T cell activation, as CXCR6 is drastically upregulated following priming by APCs (189, 199, 200).

In EAE, a study of a fate mapper and reporter model for GM-CSF production discovered that cells that are or had previously produced GM-CSF are marked with a unique gene expression profile, and that CXCR6 is highly correlated with the encephalitogenic GM-CSF-producers in the CNS (51). This finding is supported by another recent study by Hou et al. (201), which also identifies CXCR6 to be a marker of GM-CSF- and IFNγ-expressing Th cells which may also secrete IL-17A, lending support to the potential of CXCR6 as a marker for the pathogenic Th17/exTh17 subsets. Furthermore, a recent study demonstrated that SLAMF6^+^ Th17 cells in secondary lymphoid organs and intestines represent a stem-like Th17 population that convert to CXCR6^+^ GM-CSF-secreting pathogenic Th cells that invade the CNS during EAE (202). The specific role of CXCR6 in recruiting Th17 cells into the CNS during EAE is not currently clear. *Cxcr6^-/-^* mice exhibited similar clinical EAE scores to WT mice, seemingly indicating that CXCR6 is not a critical factor for EAE despite being expressed on pathogenic GM-CSF-producing Th17 cells (196). This finding contrasted somewhat with two studies which used neutralising antibodies against CXCL16 and CXCR6, which both reported impairment in the generation and recruitment of MOG_35-55_-primed T cells into the CNS and thus resulted in a decrease in severity of EAE (201, 203). Future studies that specifically address the cell-intrinsic role of CXCR6 in Th17 trafficking to the CNS in EAE are required to resolve these issues. Interestingly, some studies have questioned the potency of CXCR6 in mediating chemotaxis to CXCL16, possibly indicating that CXCR6 is not involved in migration of pathogenic cells into the CNS (196, 200), which raises the possibility that other activities of CXCL16, such as its LDL scavenging function, may instead contribute to EAE pathogenesis. Future studies that specifically address these questions will be required to elucidate the function of CXCR6 on inflammatory Th cells during EAE.

### CXCR4 and ACKR3

CXCR4 and its sole ligand, CXCL12, also play complex roles in EAE and have been implicated in inflammatory and regulatory T cell trafficking to the CNS (204). CXCR4 is expressed on a broad range of cells including T cells, monocytes, and endothelial cells (205, 206), while CXCL12 is also widely produced in multiple tissues including the brain and the bone marrow (109). Interestingly, CXCR4 has recently been identified to be part of a core Th signature, co-expressed with GM-CSF, that is associated with MS (207), which strongly implicates this receptor in pathogenic T cell homing. In addition to CXCR4, CXCL12 also binds an atypical chemokine receptor, ACKR3 (208). ACKR3 functions as a scavenger of CXCL12, creating CXCL12 gradients to modulate migration, and can form heterodimers with CXCR4 to modulate its activity (209, 210). The CXCR4-CXCL12-ACKR3 axis have been implicated in a variety of diseases including inflammatory diseases and cancer (206, 211).

In non-inflamed brains, the CXCR4-CXCL12-ACKR3 axis is involved in inducing the survival, differentiation, and migration of neuronal cells (212–214). This has led to theories that this axis could possibly be involved in remyelination (215). CXCL12 is increased in the CSF of MS patients compared to healthy controls and produced by astrocytes in response to IL-1β and MBP (216). Using immunohistochemical analysis, studies have shown that CXCL12 is constitutively expressed by endothelial cells on the abluminal surface of the BBB, but that CXCL12 production during MS shifts to the luminal site of the endothelium and this change is linked to increased MS severity (101, 217, 218). In EAE studies, this has been shown to be due to increased ACKR3 expression at abluminal sites, which caused an increased scavenging of CXCL12, although a recent study attributes this redistribution of CXCL12 to the actions of IL-20Rβ signalling in the CNS (219, 220). Recently, a study using neural network-based computer learning algorithms on MS patients identified CXCR4 expression to be a signature of the GM-CSF-expressing T cell population (207). These studies thus propose an important involvement of the CXCR4/CXCL12/ACKR3 axis during EAE/MS.

Absence of CXCR4, CXCL12 or ACKR3 are fatal to mice due to defects in embryonic development, which has limited analysis of the role of these molecules in EAE using knock-out models. Instead, antagonists of CXCR4/CXCL12/ACKR3 have been widely used. Administration of CXCR4 antagonists reduced disease severity and delayed onset of EAE (157, 221). In contrast, a CXCL12-globulin fusion protein, which acts as a CXCR4 agonist, reduced EAE severity associated with increased regulatory T cell recruitment (222). Furthermore, administration of a small molecule CXCR4 antagonist, AMD3100, resulted in an exacerbation of EAE scores, which was attributed to a loss of CXCL12 function in retaining CXCR4^+^ mononuclear cells to the perivascular space and limiting their entry into the CNS parenchyma (101). Interestingly, AMD3100 is also an agonist for ACKR3, and thus serve as both an antagonist and an agonist for two molecules of the same pathway (223). As such, the contributions of ACKR3 to pathogenic T cell trafficking EAE may also be significant. Specific blockade of ACKR3 function using a small molecule antagonist attenuated EAE due to reduced parenchymal leukocyte infiltration (219). Recently, a study using a CXCR7 antagonist resulted in a significant decrease in pathogenic Th17 levels in the CNS, and a reduced disease severity. The study postulates that CXCR7 blockade increased circulating CXCL12 levels, hence sequestering CXCR4^+^ T cell in the CNS (224). Taken together, the decrease in CXCL12 due to the increased scavenging role of ACKR3 may be important for leukocyte infiltration during EAE as CXCL12 constrains leukocytes from entering the parenchyma. This could thus explain why AMD3100 exacerbated EAE unlike other CXCR4 antagonists which do not activate ACKR3 signalling. The CXCR4/CXCL12/ACKR3 axis is thus an attractive target to investigate during EAE and may significantly impact infiltration of pathogenic T cells. Higher levels of CXCL12 functionality brought about by disrupting ACKR3 may reduce inflammatory T cell trafficking in EAE, although this remains to be formally demonstrated.

One challenge in targeting chemokine receptors as a treatment for inflammatory disease is that these same receptors are commonly used by regulatory components of the immune response such as Tregs. Indeed, Treg function often necessitates co-localisation with effector T cells (225). To do this Tregs will often express the same chemokine receptors as effector T cells. Tregs also express majority of the chemokine receptors mentioned in this review due to their requirement of entering areas of inflammation to suppress excessive inflammation. Targeting these chemokine receptors would also thus potentially inhibit Tregs from being able to enter the CNS and thus be unable to alleviate the inflammatory response (117, 138). Our laboratory has shown that specific deletion of CCR6 in T cells caused a delayed in EAE but ultimately led to a more severe course of disease, whereas deletion of CCR2 in T cells reduced overall disease severity without affecting onset of disease (111). It is therefore likely that different chemokine receptors differentially affect trafficking of Tregs and inflammatory T cells into the inflamed CNS and the evidence suggests that Treg migration relies more on CCR6 than CCR2. Therefore, if migratory receptors can be identified that are highly constrained to the pathogenic T cell subsets, this may minimise the impact on Treg migration and thus allow for a therapeutic avenue for EAE/MS. Further, more detailed profiling of multiple migratory receptors expressed by pathogenic T cells and Tregs in distinct compartments and at different time-points in CNS autoimmunity is therefore critical to determining these targets.

### CCR9

Recently, CCR9 has been shown to direct trafficking of Th17 during EAE. A study using *Tlr4* ^-/-^ mice showed that CCR9 and its ligand CCL25 were specifically decreased and the migration of Th17 cells to the CNS of *Tlr4* ^-/-^ mice was also negatively affected (226). In support of these findings, Kadowaki et al. reported that most of the CD4^+^ T memory cells in the CNS at early timepoints are indeed CCR9^+^ (227). However, these cells also expressed the co-inhibitory molecule LAG3 during the later phase of EAE, and an increase in these cells ultimately reduced severity of EAE, indicating that CCR9^+^ Th cells may have regulatory function during EAE. This would be consistent with studies of Th17 cells in murine colitis models (228). Therefore, it appears that CCR9 may have multifaceted roles during EAE and may be involved in trafficking pathogenic Th subsets early during EAE, and either Tregs or exTh17/Tregs enter during the late stages of EAE, however the definitive cell-intrinsic studies remain to be done to determine this definitively.

## Discussion and Future Perspectives

Much has been discovered over the years of research into how Th cells are able to traffic into the CNS parenchyma during MS. Several key questions regarding the mechanisms guiding T cell recruitment into the inflamed CNS remain unanswered. Impairment of a single chemokine-chemokine receptor interaction or adhesion molecule interaction is demonstrably not sufficient to completely inhibit CNS infiltration of autoreactive Th cells. This is likely due to the partially redundant expression of multiple migratory and adhesion molecules that can compensate for the lack of a single axis. These same migratory mechanisms are also used by regulatory cells to enter the CNS to some extent too. This highlights the importance of selecting receptors that are predominately required by pathogenic T cells and sparingly used by Tregs to infiltrate the CNS. The nature of the pathogenic T cell subsets that drive autoimmune inflammation and how they can be discriminated from regulatory cells based on their migratory requirements is therefore a key issue. Th17 cells and exTh17 cells have been implicated as the T cell subsets that are predominantly responsible for driving EAE through the production of GM-CSF (35, 229). However, the discovery of T cells dedicated to GM-CSF production that do not appear to emerge from Th17 or Th17 cells raises the possibility that multiple distinct GM-CSF-secreting T cell subsets contribute to autoimmune inflammation (51, 207).

Currently, the ThGM subset is associated with two chemokine receptors: CXCR6 and CXCR4 (51, 207). CXCR6 expression could conceivably promote recruitment of these cells into the CNS as astrocytes and glial cells upregulate CXCL16 during EAE. However, studies have shown that CXCR6 is not required for T cell entry into the CNS (196, 200). It is therefore likely that CXCR6 acts either in combination with other migratory receptors or does not directly contribute to recruitment to the CNS. CXCR4 is known to promote infiltration of CXCR4-expressing cells into the perivascular space of the CNS but functions to restrict entry into the CNS parenchyma. This could mean that CXCR4 activity on GM-CSF-secreting T cells prevents neuroinflammation rather than promoting infiltration of these cells. However, this remains to be elucidated. Therefore, there is a clear need for better understanding of the specific role of CXCR6 and CXCR4 with respect to recruitment of pathogenic T cells to CNS and for more detailed understanding of additional migratory receptors expressed by these cells.

## Concluding Remarks

The numerous mechanisms used by pathogenic T cells to enter the CNS to initiate and perpetuate inflammation are currently not well understood. Chemokine receptors, integrins and selectins are all key players in T cell motility, and combinatory targeting of specific chemokine receptors, integrins and selectins might be necessary to prevent inflammatory T cell entry into the CNS during EAE/MS. Whether ThGM cells and pathogenic Th17/exTh17 cells use similar mechanisms to enter the CNS is an area of great interest, as targeting the molecules involved in the mechanisms may thus yield a therapeutic that minimises side effects of targeting other cells. Future investigation is required to identify the key molecules that affect Th cell motility during neuroinflammation, and ultimately define the best molecular targets to limit pathogenic T cell trafficking in human autoimmune diseases such as MS.

## Author Contributions

AH wrote the first draft of the manuscript. CWH and CA contributed to writing of the manuscript and preparation of figures. SM and IC wrote and edited the manuscript. All authors contributed to the article and approved the submitted version.

## Funding

IC and SM hold NHMRC project grant funding (1163327 and 1163335). IC is supported by a senior fellowship from MS Australia.

## Conflict of Interest

The authors declare that the research was conducted in the absence of any commercial or financial relationships that could be construed as a potential conflict of interest.

## Publisher’s Note

All claims expressed in this article are solely those of the authors and do not necessarily represent those of their affiliated organizations, or those of the publisher, the editors and the reviewers. Any product that may be evaluated in this article, or claim that may be made by its manufacturer, is not guaranteed or endorsed by the publisher.
